# Epidemiological trends and risk factors of early-onset colorectal cancer in the founding BRICS countries, 1990–2023, with projections to 2050

**DOI:** 10.3389/fonc.2026.1924548

**Published:** 2026-09-15

**Authors:** Lin Zhang, Shuhua Zhuo, Xueying Wei, Lihuan Song, Sicheng Huang, Ye Xu, Yijuan Liu

**Affiliations:** 1 Department of Gastrointestinal Surgery, First Affiliated Hospital of Fujian Medical University, Fuzhou, China; 2 Department of Gastrointestinal Surgery, National Regional Medical Center, Binhai Campus of the First Affiliated Hospital, Fujian Medical University, Fuzhou, China; 3 Department of Geriatrics, The Eighth Affiliated Hospital of Sun Yat-sen University Shenzhen, Shenzhen, China; 4 Independent Researcher, Endoscope Center of the First Affiliated Hospital of Fujian Medical University, Fuzhou, China; 5 Department of Gastroenterology, First Affiliated Hospital of Fujian Medical University, Fuzhou, China; 6 Department of Gastroenterology, National Regional Medical Center, Binhai Campus of the First Affiliated Hospital, Fujian Medical University, Fuzhou, China

**Keywords:** BRICS countries, cancer epidemiology, clinical corroboration, disease burden, early-onset colorectal cancer, projections, risk factors

## Abstract

**Background:**

Early-onset colorectal cancer (EOCRC) is increasing globally and represents an emerging public health concern. The BRICS countries (Brazil, Russia, India, China, and South Africa), accounting for ~40% of the global population, lack comprehensive epidemiological characterization. This study evaluates EOCRC burden, risk factors, trends (1990–2023), and projections to 2050.

**Methods:**

Using Global Burden of Disease 2023 (GBD 2023) data, we analyzed incidence, prevalence, mortality, and disability-adjusted life years (DALYs) of EOCRC among individuals aged 15–49 years in BRICS countries from 1990–2023, stratified by age, sex, and region. Behavioral, dietary, and metabolic risk factors were assessed for DALY attribution. Decomposition analysis identified drivers of change. SHAP quantified feature importance. A Bayesian Age–Period–Cohort (BAPC) model projected trends to 2050. External corroboration was performed in a retrospective cohort of 236 CRC patients from a tertiary hospital in China using logistic and Cox regression analyses.

**Results:**

In 2023, China had the highest age-standardized incidence rate (ASIR) of EOCRC (3.83/100,000), while India had the lowest (1.26/100,000). From 1990–2023, Brazil showed the fastest ASIR increase (EAPC = 1.72), whereas China declined (EAPC =-1.19). Males consistently showed higher burden; in China, incidence in men aged 45–49 was 2.8-fold that of women. Key risk factors included low fiber intake, high red meat consumption, low milk intake, high BMI, and elevated fasting blood glucose, with strong co-occurrence. Decomposition analysis showed population growth and aging as main drivers, while epidemiological change varied, being positive in Brazil and negative for mortality in China. By 2050, India and South Africa are projected to show the largest increases, China will remain high with persistent sex disparities, and Russia will be stable or declining. In clinical corroboration, overweight/obesity was an independent risk factor (aOR = 2.14, 95% CI: 1.09–4.23), and diabetes predicted worse overall survival (HR = 4.35, 95% CI: 1.59–11.93), consistent with GBD-derived metabolic patterns.

**Conclusions:**

EOCRC burden varies across BRICS countries and will rise in most regions. Targeted prevention focusing on dietary and metabolic risks, plus early risk-stratified screening, is urgently needed, particularly in high-risk young males. The findings could help inform or guide future prevention and screening strategies.

## 1 Introduction

Colorectal cancer (CRC) is the third most commonly diagnosed malignancy and the second leading cause of cancer-related death worldwide (1). Although the incidence and mortality of colorectal cancer diagnosed after 50 years of age have declined in many high-income countries because of expanded screening and improved treatment, the incidence of early-onset colorectal cancer (EOCRC), typically defined as CRC diagnosed before the age of 50 years, continues to increase across multiple regions (2–4). According to the latest statistics, the incidence rate among people aged 50 and older has been declining by about 2% annually. In contrast, since the early 1990s, the incidence rate of EOCRC has been rising by about 1.5% annually, making it the leading cause of cancer-related deaths among young men and the second leading cause among young women, after breast cancer (5, 6). In accordance with the age grouping criteria of the Global Burden of Disease (GBD) study, this study operationally defines EOCRC as colorectal cancer diagnosed in individuals aged 15–49. Colorectal cancer is extremely rare in children aged 0–14 and is primarily associated with hereditary syndromes; its etiology differs fundamentally from that of EOCRC in the 15–49 age group. Therefore, excluding this age group will not have a substantial impact on the analysis results.

Unlike late-onset CRC, EOCRC affects individuals during their most economically and socially productive years. Because symptoms are often overlooked and routine screening is uncommon in younger adults, many patients present with advanced-stage disease at diagnosis (7, 8). Consequently, the increasing incidence of EOCRC has become an emerging public health concern with substantial clinical and socioeconomic implications.

Most evidence describing the global burden of CRC has been derived from the GBD study. However, studies specifically focusing on EOCRC remain limited and have largely emphasized global or regional averages, providing little insight into country-specific heterogeneity or recent temporal changes (9). This limitation is particularly relevant for the BRICS countries (Brazil, Russia, India, China, and South Africa), which are also emerging economies; these nations account for approximately 40% of the world’s population and are undergoing rapid demographic, nutritional, and lifestyle transitions associated with colorectal cancer risk (10, 11). Despite these shared transitions, considerable differences exist in healthcare systems, screening policies, population structure, and exposure to risk factors, suggesting that the epidemiology of EOCRC may vary substantially across BRICS countries. This multidimensional heterogeneity positions the BRICS countries as a unique “comparative laboratory” for studying EOCRC—enabling researchers to examine the divergent trajectories of the EOCRC burden across different stages of economic development, health system characteristics, screening policy environments, and risk exposure patterns within a single analytical framework.

This analysis exclusively enrolled the five founding BRICS members (Brazil, Russian Federation, India, China, and South Africa) rather than including newly expanded BRICS economies. The six additional member states admitted in January 2024—Saudi Arabia, Egypt, Ethiopia, Iran, the United Arab Emirates, and Indonesia—lack consistent, long-term stratified demographic and cancer surveillance data prior to 2024, accompanied by inconsistent quality of population-based cancer registries. By contrast, the five founding BRICS nations possess standardized, complete registry datasets spanning the entire study period from 1990 to 2023. Furthermore, the founding BRICS countries share highly congruent socioeconomic transitions, including rapid urbanization, progressive westernization of dietary patterns, rising prevalence of metabolic disorders, and immature nationwide colorectal cancer screening systems, forming a naturally comparable cohort for cross-national EOCRC epidemiological evaluation. Newly expanded BRICS members exhibit stark heterogeneity in population composition, staple food cultures, and national cancer screening policies, which would introduce substantial confounding factors for cross-country comparative analysis; thus, they were excluded from the present study.

Compared with prior population-level colorectal cancer analyses based on earlier iterations of the GBD database, the current work has three distinct novel contributions. First, this study specifically targets early-onset colorectal cancer in individuals aged 15–49 years, instead of adopting a broad all-age CRC perspective that dilutes the unique epidemiological features of young-onset disease. Second, we conduct a systematic cross-national burden comparison across five major emerging economies with analogous nutritional and lifestyle transition backgrounds, a comparative framework rarely reported in existing GBD-based research. Third, the study integrates two layers of evidence: population-wide disease burden quantification from GBD 2023, and individual-level clinical correlation analysis focusing on metabolic risk factors via a real-world hospital cohort, which provides triangulated evidence unavailable in purely ecological GBD reports.

To address this gap, we used GBD 2023 data to characterize the burden of EOCRC across the BRICS countries from 1990 to 2023, evaluate major modifiable risk factors, and project future trends through 2050. These findings may provide evidence to support country-specific prevention strategies and inform public health policy for EOCRC control.

To comprehensively characterize EOCRC burden and validate population-level risk associations at the individual clinical level, the present study adopts a two-component analytical framework. The primary component consists of a temporal epidemiological analysis of incidence, prevalence, mortality, and DALY metrics across the five founding BRICS countries from 1990 to 2023 using GBD 2023 data, alongside risk factor attribution and long-term disease burden projection. The secondary component is a retrospective single-center clinical cohort of Chinese CRC patients, which independently verifies the associations between metabolic abnormalities and EOCRC susceptibility and survival outcomes, supplementing the ecological limitations of aggregate GBD population data.

## 2 Methods

### 2.1 Data sources

Data were obtained from the Global Burden of Disease (GBD) 2023 study, which provides estimates for 375 diseases and injuries across 204 countries and territories between 1990 and 2023. Detailed descriptions of the GBD methodology have been published previously (12–15). The GBD 2023 dataset is publicly available through the Global Health Data Exchange (GHDx) platform (https://ghdx.healthdata.org and https://ghdx.healthdata.org/gbd-2023).

For this study, we extracted data for the five BRICS countries. Analyses included the age-standardized incidence rate (ASIR), age-standardized prevalence rate (ASPR), age-standardized mortality rate (ASMR), and age-standardized disability-adjusted life-year rate (ASDR) for colorectal cancer (CRC) among individuals aged < 50 years. Corresponding 95% uncertainty intervals (95% UI) were obtained for all estimates.

GBD estimates are generated using standardized procedures for data collection, harmonization, and statistical modeling, as described elsewhere (16). CRC was identified according to the International Classification of Diseases, 10th Revision (ICD-10), using codes C18–C21, D01.0–D01.2, and D12–D12.8 (17). Early-onset colorectal cancer (EOCRC) was defined as CRC diagnosed before 50 years of age.

### 2.2 Statistical analysis

#### 2.2.1 Trend analysis

Temporal trends in the age-standardized incidence rate (ASIR), age-standardized prevalence rate (ASPR), age-standardized mortality rate (ASMR), and age-standardized disability-adjusted life-year rate (ASDR) were evaluated using the Estimated Annual Percentage Change (EAPC). EAPC was estimated by fitting a log-linear regression model:


lnASR = α + βX + ϵ

where *ASR* represents the age-standardized rate, *X* denotes calendar year, and *β* is the regression coefficient. EAPC was calculated as:


EAPC=100×[exp(β)−1]

The corresponding 95% confidence intervals (CIs) were derived from the regression model. An ASR was considered to show an increasing trend when both the EAPC estimate and the lower limit of its 95% CI were >0, and a decreasing trend when both the EAPC estimate and the upper limit of its 95% CI were <0. Otherwise, the trend was considered stable (18).

#### 2.2.2 Decomposition analysis

To quantify the factors contributing to changes in EOCRC mortality between 1990 and 2023, total mortality change was decomposed into three components: population growth, population aging, and epidemiological change (19).

#### 2.2.3 Risk factor analysis

Ten modifiable EOCRC risk factors were obtained from the GBD 2023 database (17). These comprised five dietary factors (high processed meat intake, high red meat intake, low calcium intake, low dietary fiber intake, and low dairy intake), three behavioral factors (alcohol use, smoking, and low physical activity), and two metabolic factors (high body mass index and high fasting plasma glucose). Definitions of these risk factors and the methods used to estimate their attributable burden have been described previously (20). The GBD comparative risk assessment framework was used to estimate the proportion of EOCRC burden attributable to each risk factor.

#### 2.2.4 Risk factor correlation and importance assessment

Pearson correlation coefficients were calculated to evaluate correlations among EOCRC-related risk factors in individuals aged 15–49 years across the BRICS countries and were visualized using a heatmap. The relative contribution of each risk factor to EOCRC burden was further evaluated using SHapley Additive exPlanations (SHAP), which were derived from an eXtreme Gradient Boosting (XGBoost) regression model built on the risk factor exposures. XGBoost was chosen for its ability to capture nonlinear associations and interactions among risk factors. SHAP values were used to quantify feature importance, with the absolute value indicating the magnitude of contribution and the sign indicating the direction of effect on predicted EOCRC burden (21, 22).

#### 2.2.5 Disease burden projections

Future trends in ASIR, ASPR, ASMR, and ASDR were projected for the BRICS countries from 2024 to 2050 using a Bayesian age–period–cohort (BAPC) model. Model parameters were estimated using the Integrated Nested Laplace Approximation (INLA) algorithm within a Bayesian framework (23, 24).

All statistical analyses were performed using R software (version 4.4.1). A two-sided *P* < 0.05 was considered statistically significant.

### 2.3 Clinical corroboration cohort

To validate the population-based findings, we retrospectively analyzed 236 patients with colorectal cancer treated at the First Affiliated Hospital of Fujian Medical University between 11 November 2021 and 16 May 2022. The study was approved by the Institutional Review Board of the First Affiliated Hospital of Fujian Medical University (Approval No. [2015]084-3). The requirement for informed consent was waived because of the retrospective study design, and all data were anonymized before analysis. Patients were followed up via outpatient medical records and telephone interviews. Overall survival was defined as the interval from surgery to all-cause death or the last follow-up.

Eligible patients had a histologically confirmed diagnosis of colorectal cancer (ICD-10 codes C18–C21) and complete clinical information, including age, sex, body mass index (BMI), TNM stage, and survival status. Patients were excluded if they had a history of other malignancies, incomplete data that prevented exposure or outcome assessment, or follow-up of less than 3 months unless death or disease progression occurred during that period.

Clinical variables included demographic characteristics (age and sex), metabolic factors (BMI and diabetes history), tumor characteristics (primary site, TNM stage, and histological differentiation), postoperative complications, and survival outcomes.

Associations with EOCRC were evaluated using univariable and multivariable logistic regression models. Potential confounders were adjusted sequentially, and results are presented as odds ratios (ORs) with 95% confidence intervals (CIs).

## 3 Results

### 3.1 Overall trends in EOCRC burden

**Table 1** summarizes the ASIR, ASPR, ASMR, ASDR, and EAPC for EOCRC across the BRICS countries between 1990 and 2023. Substantial variation in disease burden was observed among countries. In 2023, China recorded the highest ASIR (3.83 per 100,000; 95% UI, 2.85–5.05), whereas India had the lowest (1.26 per 100,000; 95% UI, 0.89–1.68). From 1990 to 2023, Brazil experienced the fastest increase in ASIR (EAPC = 1.72, 95% CI: 1.62–1.82), while China showed a declining trend (EAPC = −1.19, 95% CI: −1.42 to −0.95).

**Table 1:** Trends in early-onset colon and rectum cancer by the original five BRICS countries in 1990 and 2023, with EAPC from 1990 to 2023.

Location	Measure	1990	2023	1990	2023	1990–2023
		Cases (95% UI)	Cases (95% UI)	Age-standardized rate (per 100k, 95% UI)	Age-standardized rate (per 100k, 95% UI)	EAPC (95% CI)
**Brazil**	**Incidence**	1753 (1582.21 to 1937.52)	6453.47 (5727.34 to 7286.5)	1.7 (1.54 to 1.88)	3.3 (2.92 to 3.73)	1.72(1.62 to 1.82)
**China**		40595.14 (31299.24 to 49700.83)	48143.9 (35891.51 to 63366.79)	4.44 (3.45 to 5.41)	3.83 (2.85 to 5.05)	-1.19(-1.42 to -0.95)
**Global**		115189.08 (100883.67 to 128837.86)	214616.11 (184086.91 to 246814.37)	3.07 (2.7 to 3.43)	3.26 (2.8 to 3.75)	-0.22(-0.34 to -0.1)
**India**		5039.58 (3635.79 to 6625.56)	15344.19 (10811.83 to 20459.11)	0.89 (0.64 to 1.16)	1.26 (0.89 to 1.68)	0.76(0.59 to 0.94)
**Russian Federation**		3035.43 (2708.77 to 3424.31)	3836.97 (3316.56 to 4374.61)	2.66 (2.37 to 2.99)	2.63 (2.27 to 3.01)	-0.13(-0.27 to 0.02)
**South Africa**		323.78 (239.74 to 439.7)	1236.67 (898.25 to 1618.66)	1.39 (1.03 to 1.88)	2.2 (1.6 to 2.87)	1.66(1.07 to 2.25)
**Brazil**	**Prevalence**	8289.53 (7286.85 to 9473.4)	32852.46 (28183.74 to 38341.73)	8.01 (7.05 to 9.14)	16.84 (14.44 to 19.67)	2.15(2.04 to 2.27)
**China**		188933.06 (145191.94 to 230440.45)	294121.23 (219978.06 to 374129.11)	20.67 (15.98 to 25.12)	23.43 (17.51 to 29.92)	-0.28(-0.49 to -0.06)
**Global**		606867.96 (528239.36 to 698482.57)	1251050.43 (1048977.71 to 1450687.21)	16.24 (14.17 to 18.65)	19 (15.93 to 22.05)	0.14(0.05 to 0.24)
**India**		20826.91 (15526.96 to 26757.36)	71650.44 (52839.01 to 95227.07)	3.65 (2.72 to 4.68)	5.88 (4.34 to 7.81)	1.07(0.92 to 1.23)
**Russian Federation**		13298.4 (11692.14 to 15264.63)	19942.82 (16897.96 to 23349.09)	11.59 (10.19 to 13.3)	13.69 (11.58 to 16.07)	0.58(0.4 to 0.75)
**South Africa**		1517.37 (1138.01 to 2032.09)	6880.76 (5248.36 to 8944.48)	6.47 (4.87 to 8.65)	12.24 (9.37 to 15.85)	2.56(1.75 to 3.38)
**Brazil**	**DALYs (Disability-Adjusted Life Years)**	57018.12 (52714.59 to 61892.22)	158723.94 (148294.53 to 169735.56)	54.53 (50.51 to 59.04)	81.52 (76.07 to 87.31)	0.97(0.89 to 1.05)
**China**		1412579.21 (1084385.21 to 1736071.28)	972521.36 (756394.84 to 1248940.67)	150.64 (116.55 to 184.35)	78.92 (61.25 to 101.5)	-2.75(-2.99 to -2.5)
**Global**		3337151.47 (2896055.99 to 3755445.13)	4686080.09 (4063484.27 to 5383849.7)	87.23 (76.07 to 97.81)	71.49 (61.95 to 82.18)	-1.06(-1.22 to -0.91)
**India**		189002.31 (135470.46 to 249446.79)	474658.72 (341826.71 to 632968.24)	32.72 (23.51 to 43.09)	38.89 (28.02 to 51.82)	0.23(0.08 to 0.38)
**Russian Federation**		104281.96 (93069.27 to 116513.34)	105210.4 (93288.92 to 116753.87)	90.34 (80.75 to 100.8)	72.9 (64.46 to 81.12)	-0.95(-1.1 to -0.8)
**South Africa**		11253.34 (8301.85 to 15320.58)	39235.63 (29136.88 to 51472.59)	47.46 (35.13 to 64.21)	69.35 (51.58 to 90.84)	1.4(0.87 to 1.93)
**Brazil**	**Deaths**	1105.42 (1022.51 to 1197.76)	3201.95 (2994.49 to 3422.8)	1.08 (1 to 1.17)	1.63 (1.52 to 1.74)	0.98(0.9 to 1.06)
**China**		26532.08 (20512.39 to 32513.31)	19615.92 (15167 to 25260.62)	2.91 (2.27 to 3.55)	1.56 (1.21 to 2.01)	-2.64(-2.88 to -2.41)
**Global**		64421.08 (56132.59 to 72235.04)	93098.63 (80801.5 to 107032.8)	1.72 (1.5 to 1.92)	1.41 (1.23 to 1.63)	-1.03(-1.18 to -0.88)
**India**		3728.11 (2675.92 to 4911.55)	9399.6 (6786.05 to 12535.66)	0.66 (0.47 to 0.86)	0.77 (0.56 to 1.03)	0.2(0.05 to 0.36)
**Russian Federation**		2101.37 (1878.33 to 2348.57)	2157.75 (1917.59 to 2389.93)	1.85 (1.66 to 2.06)	1.47 (1.3 to 1.63)	-0.98(-1.13 to -0.83)
**South Africa**		223.11 (164.93 to 302.38)	774.42 (576.06 to 1017.14)	0.97 (0.72 to 1.3)	1.38 (1.03 to 1.81)	1.36(0.8 to 1.92)

ASPR generally mirrored the incidence trend. The largest increases were observed in South Africa(EAPC = 2.56) and Brazil (EAPC = 2.15), whereas China showed a modest decline (EAPC = −0.28). Mortality and DALY rates also differed substantially across countries. China experienced the greatest reductions in both ASMR (EAPC = −2.64) and ASDR (EAPC = −2.75). Brazil and South Africa showed increasing trends across all four burden indicators, with South Africa exhibiting the fastest growth in mortality (EAPC = 1.36) and DALYs (EAPC = 1.40). India showed modest increases across all four burden indicators, while the Russian Federation displayed mixed trends (stable incidence, increasing prevalence, declining mortality and DALYs).

### 3.2 Age- and sex-specific patterns of EOCRC burden

**Figure 1** (and **Supplementary Table 1**) summarize the age- and sex-specific distribution of EOCRC burden in the BRICS countries and globally. Across all BRICS countries, males consistently had higher incidence, prevalence, mortality, and DALY rates than females throughout the 15–49-year age range. The magnitude of these sex differences increased with age.

**Figure 1:**
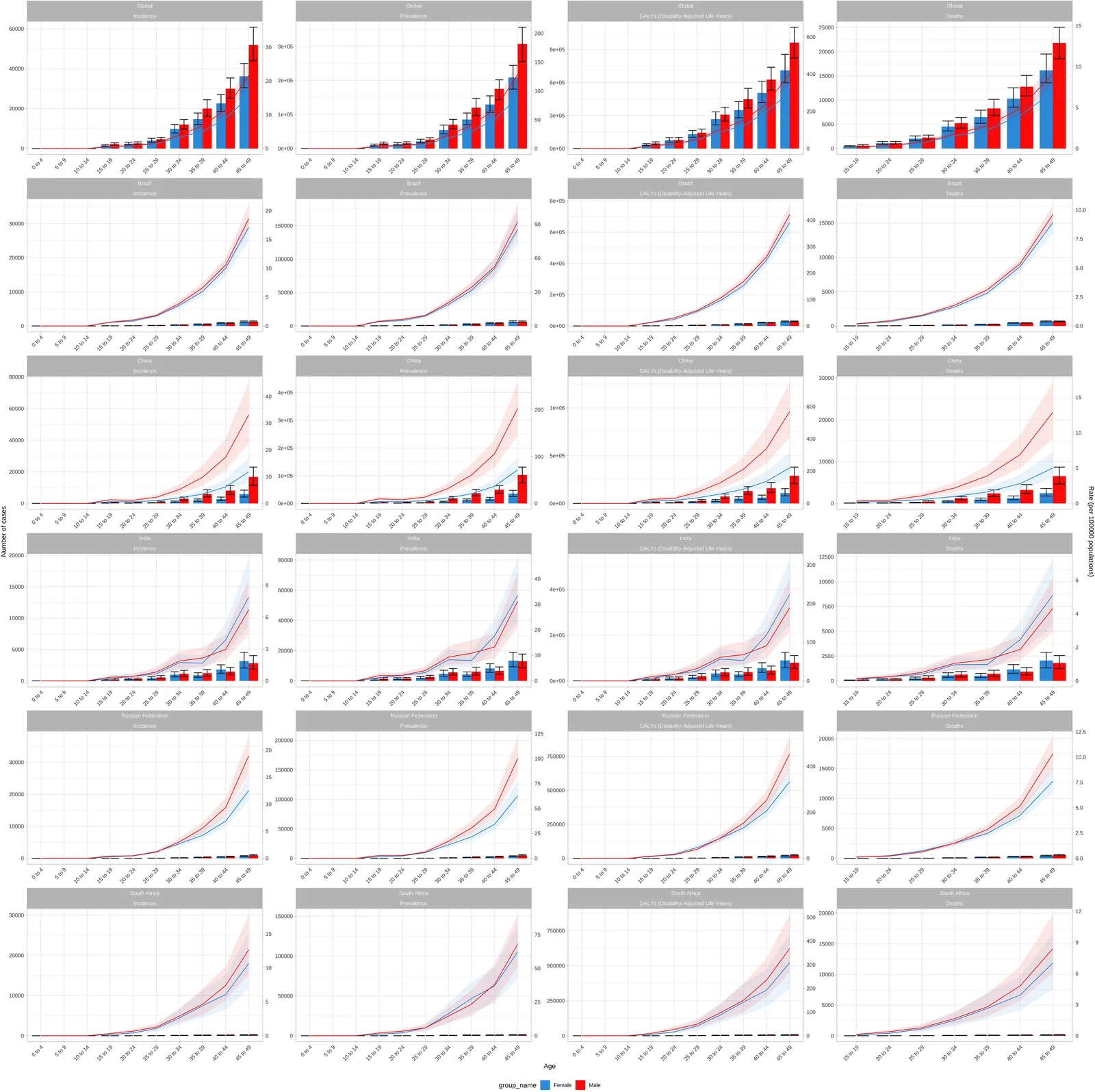
Age distribution of early-onset colorectal cancer cases across the BRICS countries and globally (by gender).

China showed the largest sex disparity in the 45–49-year age group. The male incidence rate reached 33.16 per 100,000 (95% UI: 22.57–45.36), compared with 11.84 per 100,000 (95% UI: 8.20–17.15) in females, representing a 2.8-fold difference. Male mortality was 12.88 versus 5.01 per 100,000 in females, while the DALY rate was 569.31 versus 220.60 per 100,000, corresponding to approximately 2.6-fold higher values in males. Similar age- and sex-specific patterns were observed in Brazil, India, South Africa, and the Russian Federation.

Disease burden increased markedly with age in all countries. For example, the global incidence among males aged 45–49 years was 21.71 per 100,000, approximately 33 times higher than that among those aged 15–19 years (0.66 per 100,000). Within individual age groups, males also consistently had higher DALY rates than females. In China, the DALY rate among males aged 30–34 years was 128.24 per 100,000, compared with 58.09 per 100,000 in females.

Overall, the highest EOCRC burden was observed among males aged 40–49 years across the BRICS countries.

### 3.3 Risk factor analysis

**Figure 2** shows the proportional contribution of ten risk factors to EOCRC-related DALYs globally and across the BRICS countries in 2023. Overall, dietary and metabolic factors accounted for the largest share of the attributable burden. Globally, low milk intake (18.1%), high red meat consumption (14.3%), and high BMI (10.2%) were the three leading contributors. Risk factor profiles differed across countries. In China, the largest contributors were low milk intake (21.0%), high processed meat consumption (19.1%), and alcohol use (12.8%). In Brazil, high processed meat consumption and alcohol use contributed the greatest proportion of DALYs, whereas low fiber intake and low milk intake ranked highest in India. South Africa and the Russian Federation displayed distinct risk factor profiles, indicating substantial variation in risk attribution across the BRICS countries.

**Figure 2:**
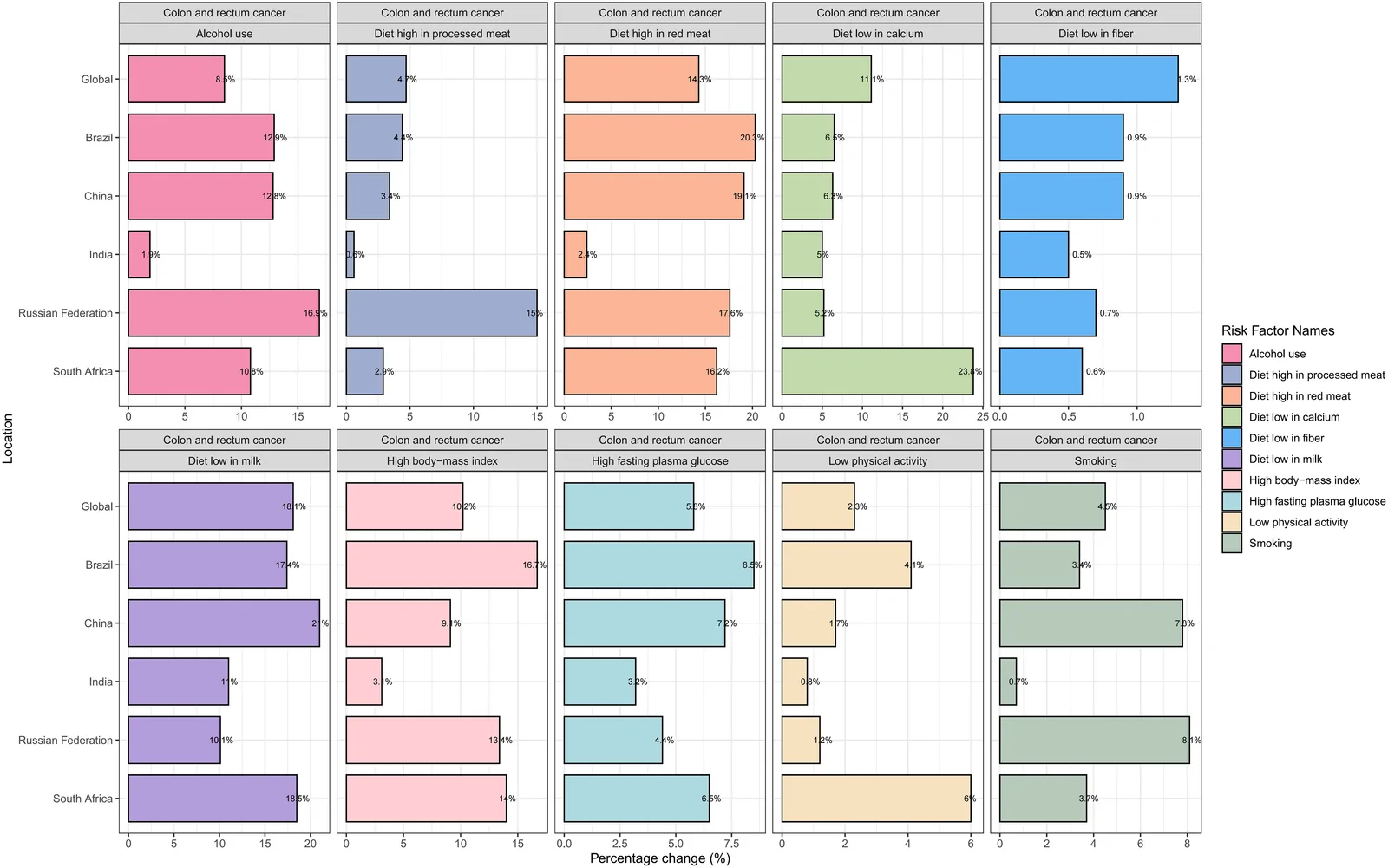
Contribution percentages of ten risk factors to the global and BRICS countries’ EOCRC disability-adjusted life years (DALYs) in 2023.

**Figure 3A** presents the pairwise correlations among EOCRC-related risk factors in individuals aged 15–49 years across the BRICS countries. Several dietary, metabolic, and behavioral factors showed strong positive correlations, suggesting that these exposures frequently coexisted. The strongest correlations were observed between low calcium intake and low physical activity (r = 0.98), low milk intake and low calcium intake (r = 0.98), and high BMI and low milk intake (r = 0.97). Alcohol consumption was also positively correlated with smoking (r = 0.73). Other notable associations included high processed meat intake with low fiber intake (r = 0.97), high BMI with high processed meat intake (r = 0.96), and high fasting blood glucose with high BMI (r = 1.00).

**Figure 3:**
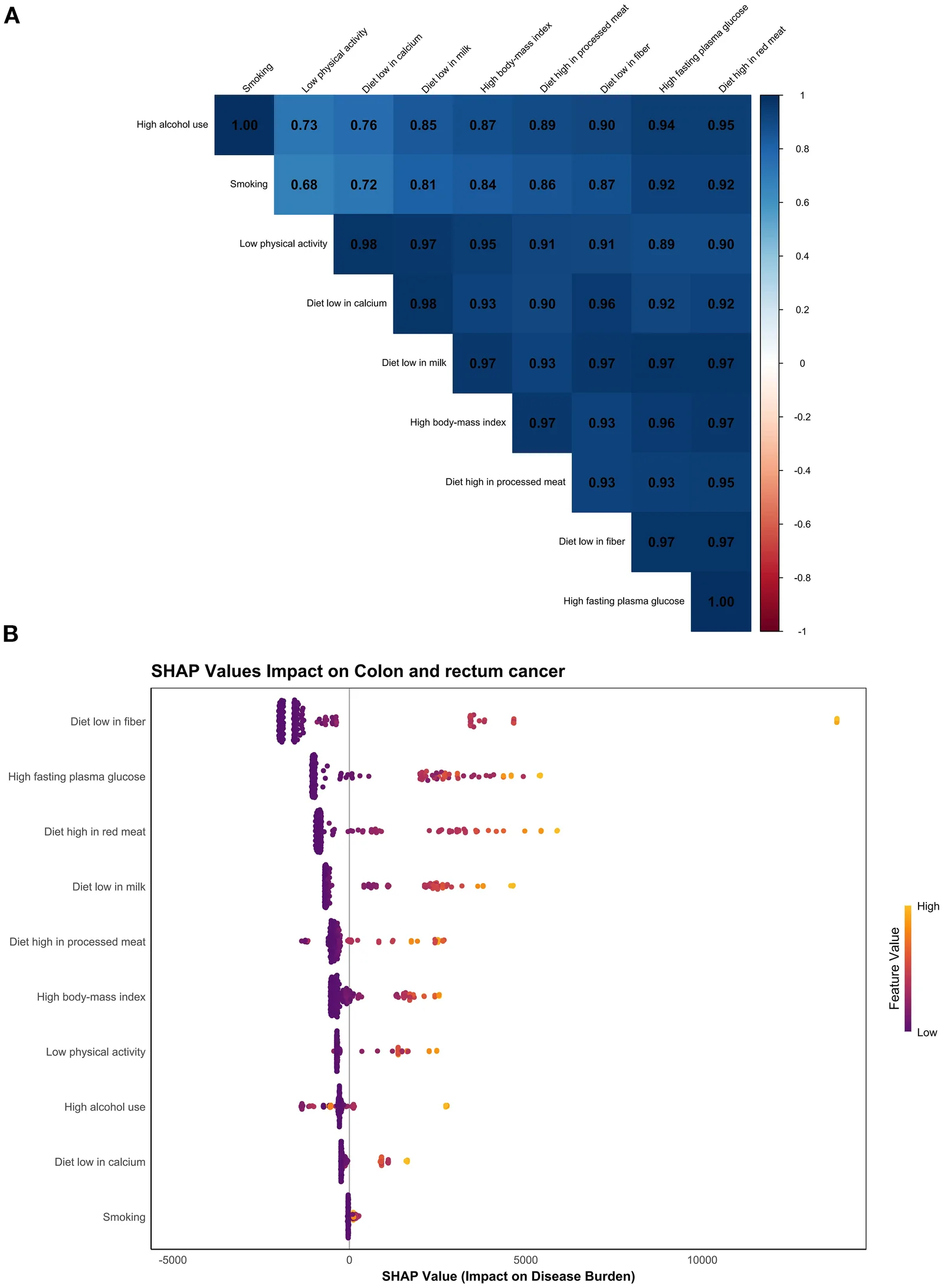
**(A)** Pairwise correlations between EOCRC-related risk factors in the 15–49 age group across the BRICS countries. **(B)** SHAP swarm plot displaying the importance ranking of various risk factors in predicting the disease burden of early-onset colorectal cancer. The color of each point represents the magnitude of the feature value (yellow for high, purple for low), while the horizontal axis of the SHAP plot reflects the direction of impact on disease burden (positive values indicate an increase in burden).

**Figure 3B** shows the relative importance of individual risk factors for EOCRC burden based on SHAP analysis. Low fiber intake was the most influential predictor, followed by high fasting blood glucose, high red meat intake, low milk intake, high processed meat intake, high BMI, low physical activity, alcohol consumption, low calcium intake, and smoking. Overall, dietary factors and metabolic factors contributed more substantially to EOCRC burden than behavioral factors.

### 3.4 Decomposition analysis

Decomposition analysis of changes in the EOCRC burden between 1990 and 2023 (**Figure 4**; **Supplementary Table 2**) showed that population growth was the main contributor to increases in incidence, prevalence, DALYs, and mortality across the BRICS countries. By contrast, the contribution of epidemiological changes (reflecting changes in age-specific rates) varied substantially across countries.

**Figure 4:**
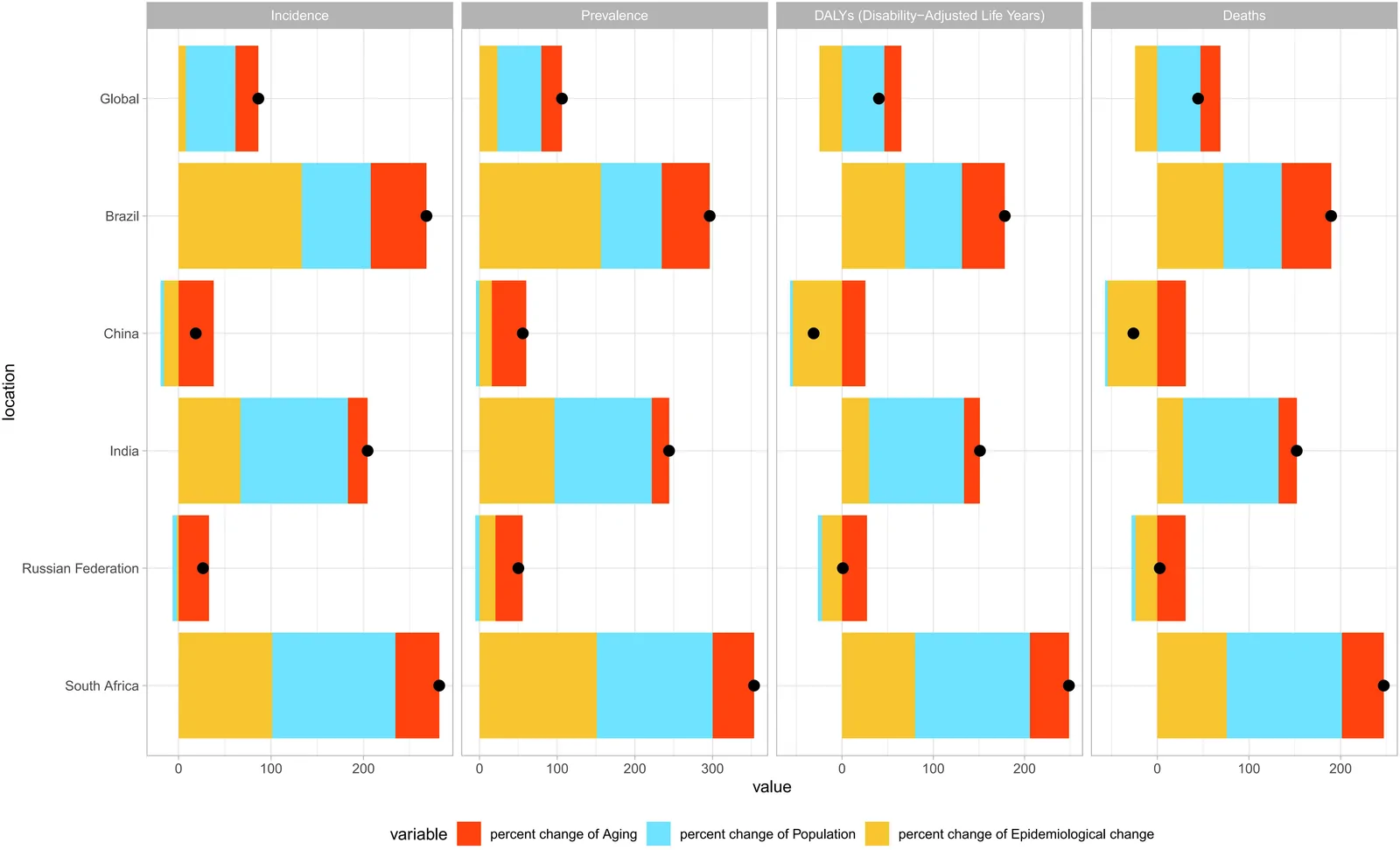
Decomposition analysis of changes in the disease burden of early-onset colorectal cancer from 1990 to 2023.

Brazil showed the largest positive contribution from epidemiological changes across all burden indicators, accounting for 133.23% of the increase in incidence. In China, epidemiological changes contributed negatively to DALYs (−53.79%) and mortality (−53.90%), partially offsetting increases attributable to population aging. In India, population growth was the predominant driver of the increasing burden, contributing 116.19% of the increase in incidence, whereas epidemiological changes made a comparatively small contribution. The Russian Federation showed little overall change in disease burden, with DALYs increasing by only 0.89%, alongside a negative contribution from epidemiological changes (−22.02%). In South Africa, epidemiological changes accounted for 101.04% of the increase in incidence.

### 3.5 Disease burden projections to 2050

BAPC model projections for EOCRC from 2024 to 2050 are presented in **Figure 5** and **Supplementary Table 3**. Overall, the projected disease burden is expected to increase globally and across most BRICS countries. India and South Africa are projected to show the largest increases in age-standardized incidence rates (ASIR) and age-standardized prevalence rates (ASPR). China is expected to maintain a high disease burden, with the ASIR projected to reach 20.2 per 100,000 (95% UI: 0–123.82) by 2050. In contrast, Russia is the only country in which most burden indicators are projected to remain stable or decline.

**Figure 5:**
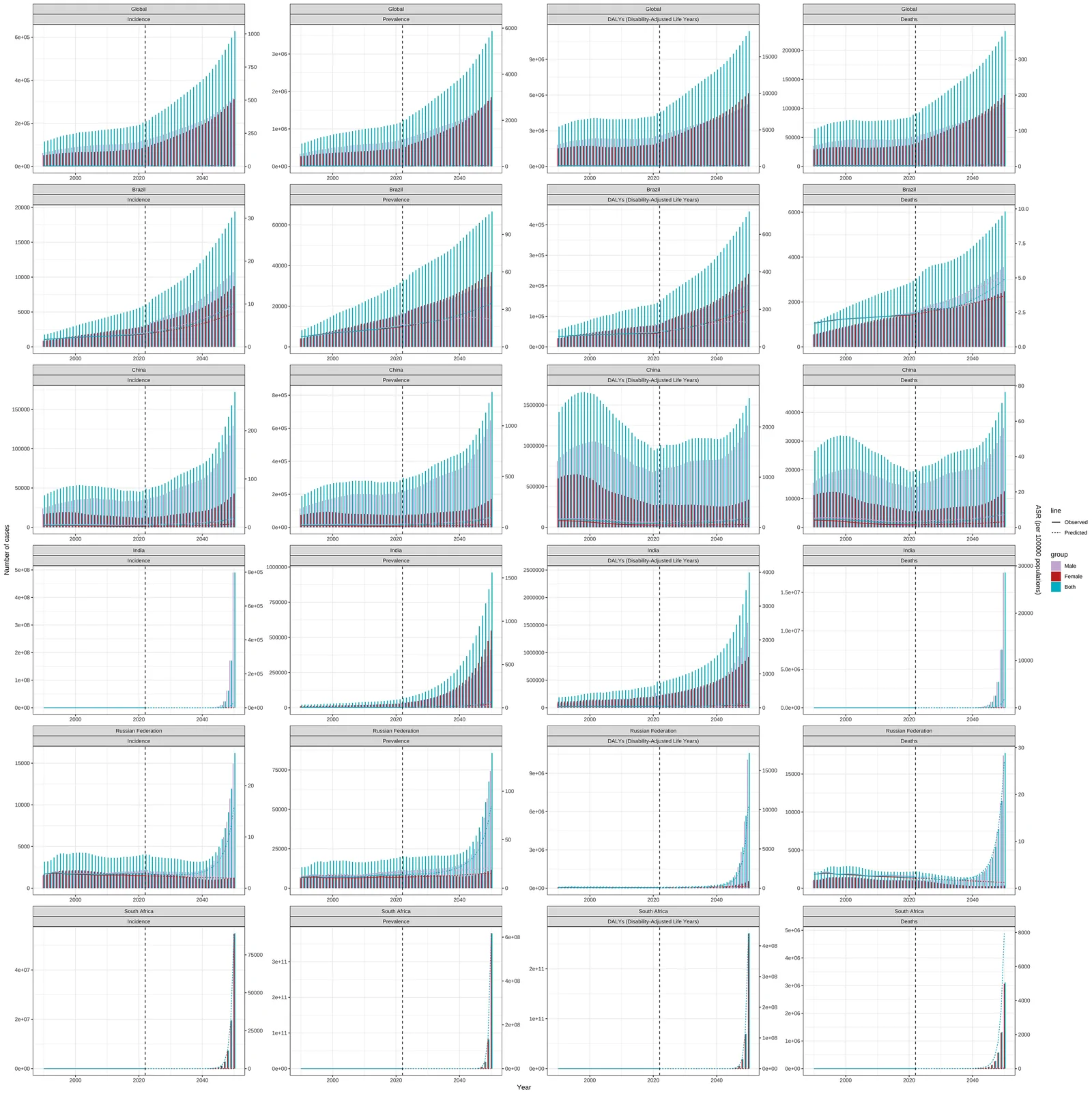
BAPC model projections for the disease burden of early-onset colorectal cancer from 2024 to 2050.

Age-standardized mortality rates (ASMR) and age-standardized DALY rates (ASDR) are projected to increase in most countries, although the projected increases are smaller than those for incidence and prevalence. Russia is also the only country in which both ASMR and ASDR are projected to decline.

Across all countries, males are projected to maintain a higher EOCRC burden than females, with the sex difference widening with increasing age. The greatest disparity is projected for China, where the male incidence rate is expected to be approximately 2.5-fold higher than the female rate by 2050.

### 3.6 Clinical corroboration

#### 3.6.1 Baseline characteristics of the clinical cohort

After applying the eligibility criteria, 236 patients with colorectal cancer were included in the retrospective clinical cohort. Of these, 48 patients (20.3%) were classified as having early-onset colorectal cancer (EOCRC; ≤50 years), while the remaining 188 comprised the late-onset group (>50 years). Baseline demographic and clinicopathological characteristics of the two groups are presented in **Table 2**.

**Table 2:** Baseline characteristics of patients with early-onset (≤50 years) versus late-onset (>50 years) colorectal cancer.

Characteristic	≤50 years (n=48)	>50 years (n=188)	P value
Demographics			
Age (years, mean ± SD)	45.6 ± 5.2	66.8 ± 10.1	<0.001
Sex, n (%)			0.516
Male	26 (54.2)	114 (60.6)	
Female	22 (45.8)	74 (39.4)	
BMI (kg/m², mean ± SD)	23.9 ± 2.7	22.8 ± 3.0	0.022
Lifestyle			
Smoking history, n (%)			0.339
No	36 (75.0)	125 (66.5)	
Yes	12 (25.0)	63 (33.5)	
Alcohol consumption, n (%)			0.344
No	35 (72.9)	121 (64.4)	
Yes	13 (27.1)	67 (35.6)	
Clinical features			
Family history of CRC, n (%)			0.017
No	44 (91.7)	186 (98.9)	
Yes	4 (8.3)	2 (1.1)	
Diabetes mellitus, n (%)			0.861
No	34 (70.8)	138 (73.4)	
Yes	14 (29.2)	50 (26.6)	
Tumour location, n (%)			0.258
Rectum	20 (41.7)	98 (52.1)	
Colon	28 (58.3)	90 (47.9)	
Pathological features			
TNM stage, n (%)			<0.001
I	20 (41.7)	28 (14.9)	
II	7 (14.6)	58 (30.9)	
III	9 (18.8)	63 (33.5)	
IV	12 (25.0)	39 (20.7)	
Pathological differentiation, n (%)			0.409
Well	1 (2.1)	1 (0.5)	
Moderate	39 (81.2)	161 (85.6)	
Poor/undifferentiated	8 (16.7)	26 (13.8)	
Treatment-related			
Surgical approach, n (%)			>0.999
Laparoscopic	46 (95.8)	181 (96.3)	
Open	2 (4.2)	7 (3.7)	
Hospital stay, days, median (IQR)	15 (11–18)	16 (12–22)	0.051
Adjuvant chemotherapy, n (%)			0.980
No	22 (45.8)	89 (47.3)	
Yes	26 (54.2)	99 (52.7)	
Postoperative complications			
Postoperative infection, n (%)			>0.999
No	36 (75.0)	140 (74.5)	
Yes	12 (25.0)	48 (25.5)	
Prognosis			
Survival status, n (%)			0.382
Alive	46 (95.8)	170 (90.4)	
Dead	2 (4.2)	18 (9.6)	

Continuous variables are presented as mean ± SD or median (IQR), and categorical variables as n (%). Comparisons were performed using the Mann–Whitney U test, χ² test, or Fisher's exact test. Two-sided P < 0.05 was considered statistically significant.Abbreviations: SD, standard deviation; IQR, interquartile range; BMI, body mass index.

#### 3.6.2 Risk factors associated with EOCRC

Univariable logistic regression identified family history of colorectal cancer and overweight/obesity as significant factors associated with EOCRC (**Table 3**). Individuals with a family history of colorectal cancer had an 8.46-fold higher odds of EOCRC (OR = 8.46, 95% CI: 1.50–47.64; P = 0.016), while overweight or obesity was associated with a 2.19-fold increase in risk (OR = 2.19, 95% CI: 1.15–4.16; P = 0.017). No significant associations were observed for sex, smoking status, alcohol consumption, or diabetes mellitus.

**Table 3:** Univariate and multivariable logistic regression analyses of risk factors for early−onset colorectal cancer (EOCRC).

Variable	Univariate analysis			Multivariable analysis		
	OR	95% CI	P value	aOR	95% CI	P value
Sex (female vs. male)	1.30	0.69–2.47	0.416	1.36	0.70–2.64	0.364
**Overweight/obesity (BMI ≥ 24 kg m^−2^)**	**2.19**	**1.15–4.16**	**0.017**	**2.14**	**1.09–4.23**	**0.027**
Smoking history (yes vs. no)	0.66	0.32–1.36	0.261	–	–	–
Alcohol consumption (yes vs. no)	0.67	0.33–1.36	0.266	–	–	–
Diabetes mellitus (yes vs. no)	1.14	0.56–2.29	0.721	0.91	0.42–1.87	0.794
**Family history of colorectal cancer (yes vs. no)**	**8.46**	**1.50–47.64**	**0.016**	**8.79**	**1.59–67.04**	**0.016**

aOR, adjusted odds ratio; CI, confidence interval. Adjusted ORs were estimated after adjustment for sex, overweight/obesity, and diabetes mellitus. Variables with P > 0.20 in the univariable analysis (smoking and alcohol consumption) were excluded from the multivariable model. Bold values indicate statistical significance (P < 0.05). Overweight/obesity was defined as BMI ≥ 24 kg/m² according to the screening standard for overweight and obesity in Chinese adults.

After adjustment for sex, diabetes mellitus, family history of colorectal cancer, and overweight/obesity, both family history (adjusted OR [aOR] = 8.79, 95% CI: 1.59–67.04; P = 0.016) and overweight/obesity (aOR = 2.14, 95% CI: 1.09–4.23; P = 0.027) remained independently associated with EOCRC (**Table 3**).

#### 3.6.3 Prognostic analysis

Multivariable Cox regression identified TNM stage IV (HR = 6.90, 95% CI: 2.52–18.93, P < 0.001) and diabetes mellitus (HR = 4.35, 95% CI: 1.59–11.93, P = 0.004) as independent predictors of poorer overall survival in patients with colorectal cancer. By contrast, overweight/obesity was not independently associated with overall survival (HR = 1.03, 95% CI: 0.39–2.72, P = 0.959) (**Figure 6**).

**Figure 6:**
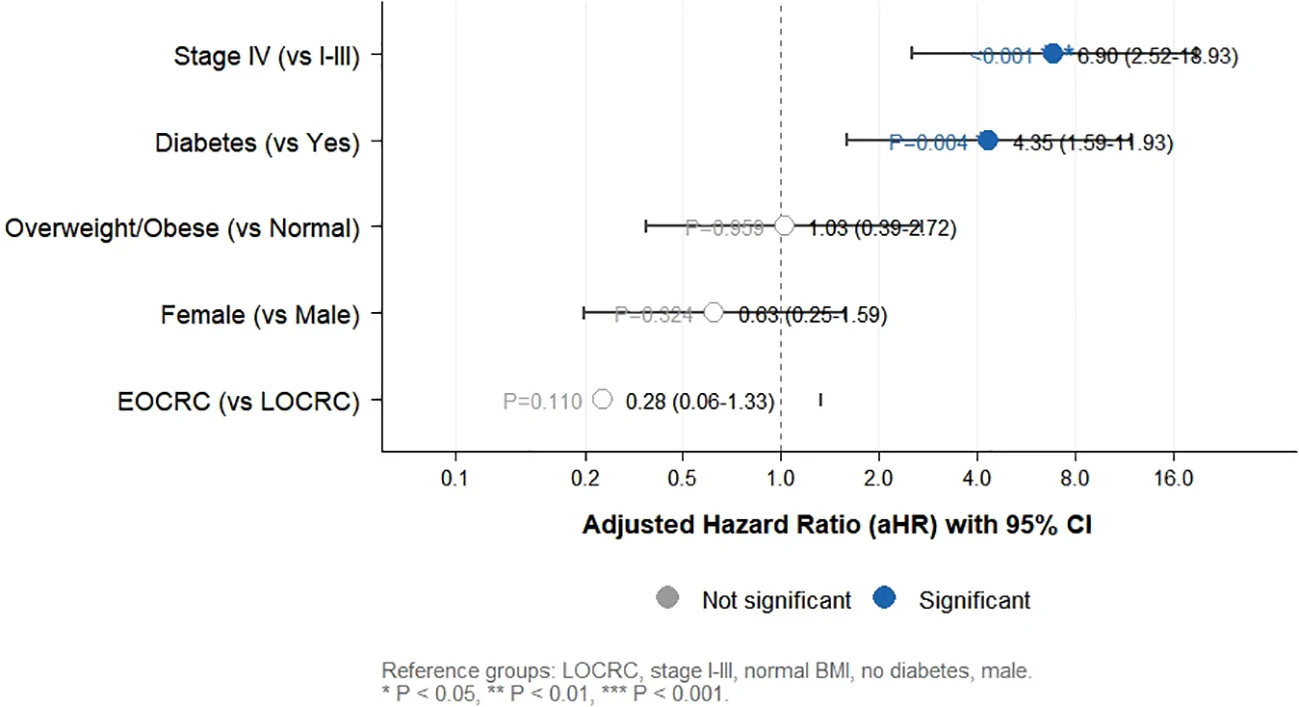
Multivariable Cox regression analysis of overall survival in patients with colorectal cancer. Forest plot showing adjusted hazard ratios (HRs) and 95% confidence intervals (CIs) derived from the multivariable Cox regression model. The model was adjusted for age group, TNM stage, body mass index (BMI), diabetes mellitus, and sex. The dashed vertical line indicates the null value (HR = 1.0). Horizontal bars represent 95% CIs. Filled dark-blue squares indicate statistically significant associations (P < 0.05), whereas hollow grey squares indicate non-significant associations. The analysis included 236 patients, of whom 20 died during follow-up.

#### 3.6.4 Summary of clinical corroboration

In the clinical cohort, men accounted for 54.2% of patients with EOCRC, although the sex difference was not statistically significant (P = 0.548). Multivariable logistic regression identified overweight/obesity (BMI ≥ 24 kg/m²) as an independent risk factor for EOCRC (adjusted odds ratio [aOR] = 2.14, 95% CI: 1.09–4.23; P = 0.027). Multivariable Cox regression further identified diabetes mellitus as an independent predictor of poorer overall survival after adjustment for age group, TNM stage, BMI, and sex (hazard ratio [HR] = 4.35, 95% CI: 1.59–11.93; P = 0.004).

The clinical corroboration cohort corroborated key findings from the GBD population-level analysis from two dimensions. First, consistent with the dominant role of metabolic factors in GBD attributable burden analysis, overweight/obesity was identified as an independent risk factor for EOCRC, and diabetes mellitus was associated with poorer survival, supporting the causal relevance of metabolic disturbances in colorectal carcinogenesis in young adults. Secondly, a higher proportion of males are found among EOCRC patients. This characteristic is consistent with the situation in the GBD dataset where the male burden of the BRICS countries is higher. These real-world clinical data provide individual-level evidence supporting the robustness of GBD 2023 estimates.

## 4 Discussion

This study offers a comprehensive assessment of the epidemiological burden, risk factors, and future trajectory of EOCRC across the BRICS countries based on GBD 2023 data covering 1990–2023. Three major findings emerged. First, the burden of EOCRC varied substantially across BRICS countries, reflecting differences in demographic change, risk-factor profiles, and national cancer control strategies. Second, dietary and metabolic risk factors consistently accounted for a large proportion of the EOCRC burden, supporting their central role in disease development. Third, BAPC projections indicate that the EOCRC burden is likely to keep growing in Brazil, India, and South Africa through 2050, whereas China will maintain a relatively high disease burden and Russia may see stable or decreasing trends, highlighting the need for strengthened prevention, early detection, and healthcare resource planning.

Marked differences in the EOCRC burden across the BRICS countries likely reflect differences in demographic transition, risk exposure, and health policy implementation. China maintained a relatively high disease burden but showed declining age-standardized incidence rates (ASIR), consistent with the expansion of screening programs and improvements in diagnosis and treatment (25). Decomposition analysis further showed that epidemiological changes offset more than half of the increase in mortality, suggesting that advances in healthcare have partly mitigated the impact of population ageing. Together, these findings support continued investment in early detection and standardized management. Brazil showed a contrasting pattern. Increases in EOCRC burden were driven primarily by epidemiological changes, consistent with rapid dietary westernization, including higher red meat intake, lower dietary fiber consumption, and increasing obesity prevalence (26, 27). These exposures have been linked to gut microbial dysbiosis, metabolic disturbances, and chronic inflammation, all of which may promote colorectal carcinogenesis (28, 29). India and South Africa experienced concurrent effects of population expansion and increasing epidemiological risk. Their relatively young and growing populations, together with rising exposure to modifiable risk factors, are likely to contribute to a sustained increase in EOCRC burden. Epigenetic alterations and immune dysregulation may partly mediate these effects (30, 31). Notably, South Africa recorded the fastest growth in prevalence, mortality, and DALY rates among BRICS countries, reflecting the superimposed effect of rapid urbanization and historically insufficient disease surveillance capacity. By comparison, the mixed trends observed in Russia—with stable incidence, increasing prevalence, and declining mortality and DALYs—may reflect a combination of established prevention efforts, improved survival leading to higher prevalence, and ongoing risk factor transitions.

This study identified dietary and metabolic factors as the major contributors to EOCRC burden at both the global and BRICS levels. Low dietary fiber intake (26, 27, 32), high red meat consumption (33, 34), low milk intake (35), elevated BMI, and high fasting blood glucose (36, 37) consistently accounted for the largest proportion of attributable burden, supporting the importance of metabolic and inflammatory pathways in EOCRC development. Several biological mechanisms may explain these associations. High red meat intake increases exposure to carcinogenic compounds, including nitrosamines, whereas inadequate dietary fiber alters gut microbial composition and reduces short-chain fatty acid production. Obesity promotes chronic inflammation through altered leptin and adiponectin signaling, while hyperglycemia activates the insulin/IGF-1 pathway (38–40). Collectively, these processes may contribute to DNA damage, epigenetic dysregulation, and aberrant epithelial proliferation, thereby facilitating colorectal carcinogenesis (2, 37, 38, 41). These mechanisms are consistent with the biological heterogeneity observed in colorectal cancer, providing biological plausibility for the epidemiological associations observed in our data.

The correlation analysis showed strong associations between several risk factors, including low calcium intake, low physical activity, low milk consumption, and high BMI (r > 0.95). These factors tend to co-occur, suggesting that they may cluster within a broader pattern of metabolic imbalance. SHAP analysis further identified low fiber intake, elevated fasting blood glucose, and high red meat consumption as the most influential variables in relation to EOCRC burden. Together, these findings indicate that risk factors are not acting in isolation, but rather in combination. From a public health perspective, this pattern suggests that single-factor interventions may have limited effectiveness. Dietary improvements such as increased whole grain and vegetable intake may improve fiber consumption and gut microbial composition (42, 43), while reducing intake of sugar-sweetened beverages and ultra-processed foods may help stabilize glycemic levels (37, 44). Regular physical activity may also improve insulin sensitivity (38, 42). Of note, the high correlation coefficients (r > 0.95) partly reflect structural collinearity inherent to the GBD estimation framework and should not be interpreted as independent biological effect sizes. These combined lifestyle modifications may offer more meaningful preventive benefits than isolated interventions.

Notably, the clinical corroboration cohort complements the GBD population-level analysis by providing individual-level evidence for risk and prognosis. GBD estimates quantify population-attributable burden but cannot establish individual exposure-outcome associations due to the ecological fallacy. Our clinical data confirmed that overweight/obesity increases EOCRC risk at the individual level, and diabetes impairs survival, bridging the gap between population-level attribution and clinical practice. Although this single-center retrospective cohort has a limited sample size, consistent metabolic associations were still detected. This triangulation of population-based big data and real-world clinical evidence strengthens the reliability of our core conclusions and provides more actionable implications for both public health policy and clinical management.

BAPC model projections suggest that EOCRC burden will continue to rise in most BRICS countries, with India and South Africa expected to experience the largest increases in incidence, potentially placing substantial pressure on healthcare systems. In China, the growth rate is projected to slow, although the absolute burden will remain high, accompanied by a marked male predominance (approximately 2.5-fold higher incidence than females). The observed gender disparity is likely multifactorial. Hormonal factors, behavioral patterns, and gut microbiota composition may all contribute. Estrogen has been suggested to exert a protective role, potentially through modulation of beneficial gut bacteria such as *Carnobacterium maltaromaticum* and vitamin D3 metabolism (45, 46). In contrast, androgens, including dihydrotestosterone, may promote colorectal carcinogenesis by altering gut microbial composition and increasing opportunistic pathogens (47, 48). Microbial metabolites such as trimethylamine and bile acids may in turn influence systemic hormone levels, suggesting a bidirectional interaction between host metabolism and microbiota (45). In addition, lifestyle-related risk factors such as alcohol consumption and smoking remain more prevalent in men and may partially explain the observed sex differences in EOCRC risk (49). These projections should be viewed as scenario-based forecasts, not deterministic predictions, because statistical models cannot fully anticipate future shifts in screening implementation, health policies, or treatment advances. Nevertheless, these findings support consideration of sex-stratified prevention strategies.

In response to these challenges and in line with the International Agency for Research on Cancer (IARC) framework for cancer prevention, we propose a stepwise prevention strategy integrating primary, secondary, and tertiary levels. At the primary prevention stage, interventions should focus on early-life exposure. This includes strengthening national nutrition policies, such as limiting the marketing of processed meat, integrating practical nutrition education into school curricula, and promoting healthy lifestyle awareness through public campaigns. Targeted education may also be needed for young men, who represent a high-risk group. For secondary prevention, screening strategies should be adapted to local healthcare capacity. In higher-resource settings, such as urban China and Brazil, cost-effectiveness analyses may support considering colonoscopy screening from age 45. In contrast, in resource-limited settings such as India and South Africa, fecal immunochemical testing (FIT) combined with risk assessment tools may offer a more feasible approach, alongside monitoring of metabolic indicators including BMI and fasting glucose in younger populations. At the tertiary level, strengthening primary healthcare remains critical, particularly to improve access to colonoscopy and minimally invasive treatment options. Given the higher burden in men, more tailored health education strategies may help improve adherence to care. Finally, expanding investment in population-based research—including molecular epidemiology, gut microbiome studies, and environmental exposure cohorts—may help clarify regional risk patterns. Improvements in cancer registry systems will also be important for supporting ongoing evaluation of prevention strategies.

This study leverages high-quality GBD 2023 data, ensuring comparability across countries, and applies a comprehensive set of analytical approaches, including trend analysis, decomposition, risk factor attribution, and predictive modeling. Together, these methods form a coherent evidence pipeline from descriptive assessment to forward projection. Several limitations should be acknowledged. First, GBD estimates are model-based and may underestimate disease burden in settings with weaker surveillance systems, particularly in rural regions of India and South Africa. In addition, risk factor exposure data are aggregated at the population level, which limits causal inference at the individual level. Both the GBD-based risk factor analyses and the clinical cohort are observational in nature, and therefore cannot be used to establish causal relationships. All findings should be interpreted within the inherent constraints of observational study designs. Second, the clinical corroboration cohort lacked detailed records of dietary intake and physical activity, so we were unable to validate the individual-level effects of the top dietary risk factors identified in the GBD analysis. Well-designed prospective cohorts collecting complete lifestyle exposure information are needed to further verify these associations. Third, The clinical cohort provided individual-level evidence supporting the metabolic risk patterns identified in the GBD analysis, although these findings are limited to a single center in China and may not be generalizable to populations with different epidemiological and healthcare profiles across BRICS countries. The consistency between population-level and individual-level data strengthens the biological plausibility of metabolic risk factors, but does not constitute cross-country corroboration. Future projections rely on historical patterns and may not fully capture the effects of emerging technologies (e.g., novel screening tools) or large-scale policy shifts (e.g., nationwide dietary interventions). Despite these constraints, the findings provide robust evidence to inform EOCRC prevention strategies in BRICS countries. Future work may focus on three directions: cross-country BRICS cohort studies to validate region-specific mechanisms; prospective intervention studies in young populations in India and South Africa evaluating low-cost strategies combining dietary modification and gut microbiome modulation; and improved predictive models incorporating policy-level variables such as screening coverage and taxation to refine burden forecasts.

In conclusion, BRICS countries are experiencing a growing burden of early-onset colorectal cancer (EOCRC), with clear heterogeneity in regional patterns and stages of development. This study provides a systematic assessment of epidemiological trends, key risk drivers, and projected disease trajectories, offering evidence to inform context-specific strategies for prevention, screening program design, and treatment. Strengthened collaboration and shared learning across BRICS countries may help improve resource allocation and support more targeted prevention approaches, which could in turn reduce the cancer burden among younger working-age populations and inform global cancer control efforts.

## Data Availability

The original contributions presented in the study are included in the article/**Supplementary Material**. Further inquiries can be directed to the corresponding authors.

## References

[1] Morgan E Arnold M Gini A Lorenzoni V Cabasag CJ Laversanne M . Global burden of colorectal cancer in 2020 and 2040: incidence and mortality estimates from GLOBOCAN. Gut. (2023) 72:338–44. doi: 10.1136/gutjnl-2022-327736 36604116

[2] Patel SG Karlitz JJ Yen T Lieu CH Boland CR . The rising tide of early-onset colorectal cancer: a comprehensive review of epidemiology, clinical features, biology, risk factors, prevention, and early detection. Lancet Gastroenterol Hepatol. (2022) 7:262–74. doi: 10.1016/s2468-1253(21)00426-x 35090605

[3] Terashima M Nakayama K Shirai S Ugai S Lee HY Matsui H . Diverging global incidence trends of early-onset cancers: comparisons with incidence trends of later-onset cancers and mortality trends of early-onset cancers. Mil Med Res. (2025) 12:79. doi: 10.1186/s40779-025-00670-8 41239501 PMC12616995

[4] Lang D Ciombor KK . Diagnosis and management of rectal cancer in patients younger than 50 years: rising global incidence and unique challenges. J Natl Compr Cancer Network JNCCN. (2022) 20:1169–75. doi: 10.6004/jnccn.2022.7056 36240852

[5] Gandini A Taieb J Blons H Netter J Laurent-Puig P Gallois C . Early-onset colorectal cancer: from the laboratory to the clinic. Cancer Treat Rev. (2024) 130:102821. doi: 10.1016/j.ctrv.2024.102821 39236404

[6] Siegel RL Giaquinto AN Jemal A . Cancer statistics, 2024. CA: A Cancer J For Clin. (2024) 74:12–49. doi: 10.3322/caac.21820 38230766

[7] Zhang L Cao F Zhang G Shi L Chen S Zhang Z . Trends in and predictions of colorectal cancer incidence and mortality in China from 1990 to 2025. Front Oncol. (2019) 9:98. doi: 10.3389/fonc.2019.00098 30847304 PMC6393365

[8] Akimoto N Ugai T Zhong R Hamada T Fujiyoshi K Giannakis M . Rising incidence of early-onset colorectal cancer - a call to action. Nat Rev Clin Oncol. (2021) 18:230–43. doi: 10.1038/s41571-020-00445-1 33219329 PMC7994182

[9] Li Q Yu M Lv H Zhang L Deng Y Yu H . Burden of early-onset colorectal cancer along with attributable risk factors from 1990 to 2019: a comparative study between China and other G20 countries. BMC Public Health. (2023) 23:1463. doi: 10.1186/s12889-023-16407-y 37525147 PMC10391986

[10] Eng C Yoshino T Ruíz-García E Mostafa N Cann CG O'Brian B . Colorectal cancer. Lancet (London England). (2024) 404:294–310. doi: 10.1016/s0140-6736(24)00360-x 38909621

[11] Burnett-Hartman AN Lee JK Demb J Gupta S . An update on the epidemiology, molecular characterization, diagnosis, and screening strategies for early-onset colorectal cancer. Gastroenterology. (2021) 160:1041–9. doi: 10.1053/j.gastro.2020.12.068 33417940 PMC8273929

[12] The global, regional, and national burden of cancer, 1990-2023, with forecasts to 2050: a systematic analysis for the global burden of disease study 2023. Lancet (London England). (2025) 406:1565–86. doi: 10.1016/s0140-6736(25)01635-6 41015051 PMC12687902

[13] Global burden of 292 causes of death in 204 countries and territories and 660 subnational locations, 1990-2023: a systematic analysis for the global burden of disease study 2023. Lancet (London England). (2025) 406:1811–72. doi: 10.1016/s0140-6736(25)01917-8 41092928 PMC12535838

[14] Global age-sex-specific all-cause mortality and life expectancy estimates for 204 countries and territories and 660 subnational locations, 1950-2023: a demographic analysis for the global burden of disease study 2023. Lancet (London England). (2025) 406:1731–810. doi: 10.1016/s0140-6736(25)01330-3 41092927 PMC12535839

[15] Burden of 375 diseases and injuries, risk-attributable burden of 88 risk factors, and healthy life expectancy in 204 countries and territories, including 660 subnational locations, 1990-2023: a systematic analysis for the global burden of disease study 2023. Lancet (London England). (2025) 406:1873–922. doi: 10.1016/s0140-6736(25)01637-x 41092926 PMC12535840

[16] Global burden of 369 diseases and injuries in 204 countries and territories, 1990-2019: a systematic analysis for the global burden of disease study 2019. Lancet (London England). (2020) 396:1204–22. doi: 10.1016/s0140-6736(20)30925-9 33069326 PMC7567026

[17] Global, regional, and national burden of colorectal cancer and its risk factors, 1990-2019: a systematic analysis for the global burden of disease study 2019. Lancet Gastroenterol Hepatol. (2022) 7:627–47. doi: 10.1016/s2468-1253(22)00044-9 35397795 PMC9192760

[18] Clegg LX Hankey BF Tiwari R Feuer EJ Edwards BK . Estimating average annual per cent change in trend analysis. Stat Med. (2009) 28:3670–82. doi: 10.1002/sim.3733 19856324 PMC2843083

[19] Pou SA Tumas N Coquet JB Niclis C Román MD Díaz MD . Burden of cancer mortality and differences attributable to demographic aging and risk factors in Argentina, 1986-2011. Cadernos Saude Publica. (2017) 33:e00016616. doi: 10.1590/0102-311x00016616 28300967

[20] Global burden of 87 risk factors in 204 countries and territories, 1990-2019: a systematic analysis for the global burden of disease study 2019. Lancet (London England). (2020) 396:1223–49. doi: 10.1016/s0140-6736(20)30752-2 33069327 PMC7566194

[21] Liu YC Liu YH Pan HF Wang W . Unveiling new insights into migraine risk stratification using machine learning models of adjustable risk factors. J Headache Pain. (2025) 26:103. doi: 10.1186/s10194-025-02049-5 40329184 PMC12057085

[22] Bifarin OO . Interpretable machine learning with tree-based shapley additive explanations: application to metabolomics datasets for binary classification. PloS One. (2023) 18:e0284315. doi: 10.1371/journal.pone.0284315 37141218 PMC10159207

[23] Knoll M Furkel J Debus J Abdollahi A Karch A Stock C . An R package for an integrated evaluation of statistical approaches to cancer incidence projection. BMC Med Res Methodol. (2020) 20:257. doi: 10.1186/s12874-020-01133-5 33059585 PMC7559591

[24] Riebler A Held L . Projecting the future burden of cancer: Bayesian age-period-cohort analysis with integrated nested Laplace approximations. Biometrical J Biometrische Z. (2017) 59:531–49. doi: 10.1002/bimj.201500263 28139001

[25] Gao XH Li J Liu LJ Zheng NX Zheng K Mei Z . Trends, clinicopathological features, surgical treatment patterns and prognoses of early-onset versus late-onset colorectal cancer: a retrospective cohort study on 34067 patients managed from 2000 to 2021 in a Chinese tertiary center. Int J Surg (London England). (2022) 104:106780. doi: 10.1016/j.ijsu.2022.106780 35850466

[26] John Kenneth M Tsai HC Fang CY Hussain B Chiu YC Hsu BM . Diet-mediated gut microbial community modulation and signature metabolites as potential biomarkers for early diagnosis, prognosis, prevention and stage-specific treatment of colorectal cancer. J Adv Res. (2023) 52:45–57. doi: 10.1016/j.jare.2022.12.015 36596411 PMC10555786

[27] Hou H Chen D Zhang K Zhang W Liu T Wang S . Gut microbiota-derived short-chain fatty acids and colorectal cancer: ready for clinical translation? Cancer Lett. (2022) 526:225–35. doi: 10.1016/j.canlet.2021.11.027 34843863

[28] Han JX Tao ZH Qian Y Yu CY Li J Kang ZR . ZFP90 drives the initiation of colitis-associated colorectal cancer via a microbiota-dependent strategy. Gut Microbes. (2021) 13:1–20. doi: 10.1080/19490976.2021.1917269 33947304 PMC8115455

[29] Richter KM Wrage M Krekeler C De Oliveira T Conradi LC Menck K . Model systems to study tumor-microbiome interactions in early-onset colorectal cancer. EMBO Mol Med. (2025) 17:395–413. doi: 10.1038/s44321-025-00198-3 39948421 PMC11903813

[30] Zinkeng A Taylor FL Cheong SH Song H Merchant JL . Early onset colorectal cancer: molecular underpinnings accelerating occurrence. Cell Mol Gastroenterol Hepatol. (2025) 19:101425. doi: 10.1016/j.jcmgh.2024.101425 39510499 PMC11731505

[31] Hamdy NM Zaki MB Rizk NI Abdelmaksoud NM Abd-Elmawla MA Ismail RA . Unraveling the ncRNA landscape that governs colorectal cancer: a roadmap to personalized therapeutics. Life Sci. (2024) 354:122946. doi: 10.1016/j.lfs.2024.122946 39122108

[32] Zhang H Tian Y Xu C Chen M Xiang Z Gu L . Crosstalk between gut microbiotas and fatty acid metabolism in colorectal cancer. Cell Death Discov. (2025) 11:78. doi: 10.1038/s41420-025-02364-5 40011436 PMC11865559

[33] Farvid MS Sidahmed E Spence ND Mante Angua K Rosner BA Barnett JB . Consumption of red meat and processed meat and cancer incidence: a systematic review and meta-analysis of prospective studies. Eur J Epidemiol. (2021) 36:937–51. doi: 10.1007/s10654-021-00741-9 34455534

[34] Ungvari Z Fekete M Varga P Lehoczki A Munkácsy G Fekete JT . Association between red and processed meat consumption and colorectal cancer risk: a comprehensive meta-analysis of prospective studies. GeroScience. (2025) 47:5123–40. doi: 10.1007/s11357-025-01646-1 40210826 PMC12181564

[35] Nimptsch K Lee DH Zhang X Song M Farvid MS Rezende LFM . Dairy intake during adolescence and risk of colorectal adenoma later in life. Br J Cancer. (2021) 124:1160–8. doi: 10.1038/s41416-020-01203-x 33398066 PMC7960961

[36] Li H Boakye D Chen X Jansen L Chang-Claude J Hoffmeister M . Associations of body mass index at different ages with early-onset colorectal cancer. Gastroenterology. (2022) 162:1088–1097.e3. doi: 10.1053/j.gastro.2021.12.239 34914944

[37] Colloca A Donisi I Anastasio C Balestrieri ML D'Onofrio N . Metabolic alteration bridging the prediabetic state and colorectal cancer. Cells. (2024) 13. doi: 10.3390/cells13080663 38667278 PMC11049175

[38] Samuel SM Varghese E Büsselberg D . Complexity of insulin resistance in early-onset colorectal cancer. Cancer Cell. (2025) 43:797–802. doi: 10.1016/j.ccell.2025.03.033 40250447

[39] Li Y Li K Pan T Xie Q Cheng Y Wu X . Translocation of IGF-1R in endoplasmic reticulum enhances SERCA2 activity to trigger Ca(2+)(ER) perturbation in hepatocellular carcinoma. Acta Pharm Sin B. (2023) 13:3744–55. doi: 10.1016/j.apsb.2023.05.031 37719369 PMC10501870

[40] Park HH Hwang W Ham S Kim E Altintas O Park S . A PTEN variant uncouples longevity from impaired fitness in Caenorhabditis elegans with reduced insulin/IGF-1 signaling. Nat Commun. (2021) 12:5631. doi: 10.1038/s41467-021-25920-w 34561453 PMC8463539

[41] Zhu Y Li X . Advances of Wnt signalling pathway in colorectal cancer. Cells. (2023) 12. doi: 10.3390/cells12030447 36766788 PMC9913588

[42] Markozannes G Becerra-Tomás N Cariolou M Balducci K Vieira R Kiss S . Post-diagnosis physical activity and sedentary behaviour and colorectal cancer prognosis: a Global Cancer Update Programme (CUP Global) systematic literature review and meta-analysis. Int J Cancer. (2024) 155:426–44. doi: 10.1002/ijc.34903 38692650

[43] Zheng X Hur J Nguyen LH Liu J Song M Wu K . Comprehensive assessment of diet quality and risk of precursors of early-onset colorectal cancer. J Natl Cancer Institute. (2021) 113:543–52. doi: 10.1093/jnci/djaa164 33136160 PMC8096368

[44] Joh HK Lee DH Hur J Nimptsch K Chang Y Joung H . Simple sugar and sugar-sweetened beverage intake during adolescence and risk of colorectal cancer precursors. Gastroenterology. (2021) 161:128–142.e20. doi: 10.1053/j.gastro.2021.03.028 33753105 PMC8238879

[45] Wu Z Huang Y Zhang R Zheng C You F Wang M . Sex differences in colorectal cancer: with a focus on sex hormone-gut microbiome axis. Cell Communication Signaling CCS. (2024) 22:167. doi: 10.1186/s12964-024-01549-2 38454453 PMC10921775

[46] Zhou YL Hong J . Gut microbiota: guardians of the female gut health. Cancer Cell. (2023) 41:1392–4. doi: 10.1016/j.ccell.2023.07.004 37541242

[47] Wang L Tu YX Chen L Zhang Y Pan XL Yang SQ . Male-biased gut microbiome and metabolites aggravate colorectal cancer development. Advanced Sci (Weinheim Baden-Wurttemberg Germany). (2023) 10:e2206238. doi: 10.1002/advs.202206238 37400423 PMC10477899

[48] Li Y Jiang HL Chen L Yu JY Aryal S Gao YN . Sex hormone androgen elevates blood pressure through gut microbiota-TMAO pathway. Hypertension (Dallas Tex 1979). (2025) 82:1999–2011. doi: 10.1161/hypertensionaha.125.25052 40832683 PMC12703944

[49] Jin EH Han K Shin CM Lee DH Kang SJ Lim JH . Sex and tumor-site differences in the association of alcohol intake with the risk of early-onset colorectal cancer. J Clin Oncol Off J Am Soc Clin Oncol. (2023) 41:3816–25. doi: 10.1200/jco.22.01895 37315287 PMC10419447

